# Health related quality of life and buffering factors in adult survivors of acute pediatric lymphoblastic leukemia and their siblings

**DOI:** 10.1186/s12955-021-01700-4

**Published:** 2021-02-12

**Authors:** Katarina Aili, Susann Arvidsson, Jens M. Nygren

**Affiliations:** 1 School of Health and Welfare, Halmstad University, Halmstad, Sweden; 2 Institute of Environmental Medicine, Karolinska Institutet, Stockholm, Sweden

## Abstract

**Background:**

The improvement in treatment of pediatric acute lymphatic leukemia (ALL) has introduced new challenges for pediatric oncology care in understanding and handling long-term treatment-related complications later in adult life. The aim of this study was to describe health related quality of life (HRQoL) and the relation to buffering factors among young adult (YA) pediatric ALL survivors and their siblings.

**Methods:**

This cross-sectional study was performed among 227 adults, treated for pediatric ALL in Sweden between 1985 and 1997 and their siblings (n = 70). Group means of HRQoL (SF-36) were compared between YA ALL survivors and the siblings, as well as to normative values from the general population. Self-efficacy (GSES) and social support (SS-13 subscale AVSI) was considering potential buffering factors for HRQoL and mental health. Associations between HRQoL and mental health respectively and self-efficacy and social support was analyzed.

**Results:**

The YA ALL survivors scored significantly lower on the HRQoL parameters general health (69.6 vs. 78.4, p = 0.004) and role emotional (77.1 vs. 88.1, p = 0.014), than the siblings. Further, they reported significantly lower general health (69.6 vs. 75.8), vitality (56.9 vs. 68.8), social functioning (84.5 vs. 88.6), role emotional (77.1 vs. 85.7) and mental health (71.3 vs. 80.9) compared with Swedish norms. Both YA ALL survivors and the siblings reported lower vitality and worse mental health than the general population. The HRQoL parameters, depression, stress and anxiety were all associated with both self-efficacy and social support among the YA ALL survivors. Among the siblings however, only general health, vitality, role emotional, mental health and depression were associated with social support, and only general health and mental health were associated with general self-efficacy.

**Conclusion:**

The results from this study show that buffering factors, like social support and self-efficacy, may play an important role for psychosocial outcomes and HRQoL among YA ALL survivors later in life. The results suggest that this group could benefit from continuous support in adult life to handle consequences of their pediatric disease.

## Background

Acute lymphatic leukemia (ALL) is the most common cancer in children with highest incidence among 2–4 year old’s. In many countries, the prognosis has improved substantially and continually during the last 60 years due to improved treatment protocols. This has resulted in a five-year survival rate up to 90% [1, 2] and an over-all life expectancy similar to the general population [3].

Treatment protocols have improved to reduce treatment-related complications both during treatment and later in life [4]. However, children with ALL do experience impairment in physical and mental health and in health-related quality of life (HRQoL) after completion of the up to 3-year-long treatment [5, 6]. A long-term disease burden remains decades post treatment [7]. While the long-term prevalence of physical health problems has been more widely investigated, knowledge on the long-term prevalence of mental health problems remains scarce. Results indicate a higher prevalence of mental ill health [8, 9] poorer self-image [9] and mild to severe anxiety and depression [10]. There are however studies that have found the opposite, that ALL survivors report better HRQoL healthy controls of similar age [11]. The latter study suggests a problem with report bias when assessing HRQoL among cancer survivors, and that future studies should include a wider range of measurements, e.g. assessment of coping mechanisms [11]. Among survivors of pediatric ALL a large variety in overall HRQoL has been described. The experience of low HRQoL is associated with treatment related factors, physical late effects, fatigue/insomnia, anxiety and depression [12, 13] whereas higher HRQoL is associated with protecting factors such as resiliency, coping skills, independence, and achievement of life goals [13].

The definition of HRQoL differs based on the instrument being used and for long-term follow-ups, there are advantages and disadvantages using generic respective disease specific HRQoL instruments [14]. The generic instruments may be inferior in capturing phenomena specific to a particular diagnostic group, but for long-term follow-up after pediatric ALL, where the study group is in remission and has a HRQoL comparable to the normal population, a disease-specific instrument may be less suitable, especially if the results should be compared with a control group without the specific disease history. The SF-36 is a generic scale based on eight subscales measuring different dimensions of HRQoL15. Apart from being a widely used instrument to assess HRQoL as an outcome, lower scorings on the SF-36 scale predict poor health outcomes, including development of chronic widespread pain [16], long term sickness absence [17] and mortality [18]. The late effects of pediatric ALL is often investigated from a perspective of pathology. Disorders with less distinct pathological representation, but which imposes substantial suffering for the affected, such as the mental and social late effects of previous illness, has been less thoroughly described. The siblings of survivors of pediatric ALL are sometimes used as a healthy control group, when estimating the long-term effects [19]. This is advantageous when the interest lies in separating effects of the disease and effects from the environment, such as the family [20].

Thus, the improvement in treatment of pediatric ALL has introduced new challenges for pediatric oncology care in understanding and handling treatment-related complications that requires long-term follow-up beyond treatment, remission and survival, and into adulthood. To meet these challenges, studies examining health and HRQoL post treatment are needed to support the development of care processes that identify problems early and address its consequences. Research has shown the importance of risk factors for the occurrence of HRQoL. But the care processes needed to promote HRQoL among cancer survivors should not only be based on identifying and managing risks in relation to HRQoL, but also support factors that may have a buffering effect on the HRQoL over time. Changes in social support and self-efficacy has been shown to be associated with changes in HRQoL over time in children and adolescents [21, 22], which supports the idea that these factors may be buffering also among young adults that have survived pediatric ALL. Prevention programs focusing on such buffering factors can be especially important for the HRQoL outcomes among young adult (YA) survivors of ALL where there is a high incidence of risk factors.

The aim of this study was to describe HRQoL and the relation to the potential buffering factors self-efficacy and social support among YA survivors of pediatric ALL and their siblings.

## Method

This cross-sectional study used data from a nationwide survey in Sweden 2012 including young adult survivors of pediatric ALL and their siblings in Sweden. The aim of the survey was to investigate potential long-term effects on different dimensions on health, HRQoL and comorbidities, and to assess care seeking behavior of the young adult survivors. The survey was carried out in 2012 in Sweden with approval from the regional ethical board (dnr 2010/579).

### Participants

Study participants were recruited from the Swedish Children's Cancer Register. All individuals who were diagnosed with ALL between 1985 and 1997 and that were 0–15 years old when diagnosed and more than 18 years old at the time of the study, were eligible for participation. Before 1985 radiation therapy was used in some standard protocols, and the time range was chosen in order to get a more homogenous sample regarding potential long-term physiological effects from radiation treatment.

In all, 416 individuals were identified by the register and their contact details were retrieved from the Swedish population register (SPAR, Statistics Sweden). At the time when questionnaires were sent out to the eligible participants it was between 14 and 27 years since they had gone through the ALL treatment. Participants were approached by letters including information about the study, a consent form and the 71-item questionnaire. In 42 cases, the presence of mental health problems, disabilities (downs syndrome), emigration or longer stays abroad was confided. Out of the remaining 374 individuals, 227 (61%) completed the questionnaires after up to two reminders. The siblings were recruited through the participants (by the questionnaire). In all, 110 siblings were approached for participation in the study, out of which 70 siblings agreed to participate and completed the questionnaire.

### Descriptive parameters

For descriptive purposes, comparisons between the YA ALL survivors and siblings were made on parameters that can have relevance for health.

*Physical activity* was assessed by reported amount of activity during a week. The respondents could choose from seven predefined levels of activity defined by weekly distance in walking and/or running and/or time spent with strenuous physical activity (e.g. the seven levels of running were: (1) none; (2) 1 km; (3) 2–10 km; (4) 10–15 km; (5) 15–25 km; (6) 25–40 km; (7) > 40 km). These levels were further categorized into (1) “inactive” (No activity; or running 1 km and/or walking 1 km and/or 30 min of strenuous physical activity per week); (2) “active” (running 2–15 km and/or walking 2–20 km and/or 0.5 to 4 h of strenuous activity per week); and “very active” (running > 15 km and/or walking > 20 km and/or > 4 h of strenuous activity per week). The categorization was made based on what was most probable to respond to the WHO recommendations on physical activity [23].

*Body mass index (BMI)* was calculated from self-reported height and weight by weight (in kg) divided by height (in meters) in squares (kg/m^2^), and categorized into (1) BMI of < 19; (2) BMI 19–24; (3) BMI 25–29; (4) 30–34; (5) 35–39; (6) ≥ 40.

*Comorbidity* was assessed by items in the questionnaire based on CCI, Charlson Comorbidity Index [24], including: *Cardio vascular disease (CVD)* (including items on ischemic heart disease, heart failure, bypass and stroke); *Respiratory disease* (Chronic obstructive pulmonary disease (COPD) and asthma); *Headache/migraine, Ulcer disease, Diabetes, Renal disease, Connective tissue disease* (including Rheumatoid arthritis, Systemic Lupus Erythematosus (SLE) and Polymyalgia Rheumatica), *Cancer, Liver disease* (Liver cirrhosis or severe liver damage).

In addition, *Marital status*, *Having children*, *Educational level* (by highest finished education), *Occupational status* and *Income* (monthly personal income) was assessed by the questionnaire.

The included participants and the drop-outs (those who did not respond to the questionnaire sent out) were further described by age, gender, age at diagnosis and years from diagnosis (at time of the study) and if they had received radiation therapy as part of their cancer treatment.

### Assessment of main variables

*Health related quality of life (HRQoL)* was assessed by the SF-36 questionnaire including the eight subscales (1) General Health (GH); (2) Role Emotional (RE); (3) Role Physical (RP); (4) Mental Health (MH); (5) Vitality (VT); (6) Physical Functioning (PF); (7) Bodily Pain (BP); and Social Functioning (SF) [25]. The scorings from the subscales were calculated as appropriate according the manual, were each subscale range from 0 to 100 (were a higher value indicate a better HRQoL status). The SF-36 questionnaire is a validated instrument that has been widely used for assessing HRQoL in adults, also when tested specifically for assessing HRQoL among adult childhood cancer survivors [26]. Normative values on the SF-36 subscales based on a Swedish population are available for different age groups [27] and such values for age group 25–34 years are used for comparison in this study.

*Depression, anxiety and stress* were assessed by the Depression Anxiety Stress Scales (DASS) 21 which is a short version of DASS 42 [28, 29]. Each subscale is comprised of 7 items with responses reflecting three levels. To yield equivalent scores to the DASS 42, the total score of each subscale should be multiplied by two, and thus range from 0 to 42. The sub scores are categorized according to severity into normal, mild, moderate, severe and extremely severe [28]. The DASS was chosen as a complement to the MH scale from SF-36 questionnaire to get a more precise assessment of mental health conditions and to be able to separate depression, anxiety and stress.

*Social support* and *self-efficacy* were assessed and analyzed as potential buffering factors. *Social support* was assessed by the SS-13 subscale AVSI, to describe quantitative aspects of social support, or the availability of social integration [30]. *Self-efficacy* was assessed by the general self-efficacy scale (GSES) [31, 32]. The GSES is a 10 item questionnaire, yielding a total score of 10–40, where a higher score indicate better self-efficacy.

### Data analyses

Group differences between included participants and dropouts, and between YA ALL survivors and siblings respectively, were analyzed by Mann–Whitney U-test for continuous variables, and chi-squared test for categorical variables. Differences in HRQoL, were tested by analyzing differences between groups on each subscale separately. Normative mean values and 95% confidence intervals for the subscales were assessed from the manual for SF-36 [27]. In addition, the Mann Whitney U-test for differences in scorings on the SF-36 between YA ALL survivors and their siblings were made, due to the skewed distribution of the SF-36 variables.

For associations between the hypothesized buffering factors social support and general self-efficacy, and the HRQoL parameters and the mental health parameters respectively, multiple regression analyses including age and gender as covariates were performed separately for the YA ALL survivors and the siblings. The method “enter” was used when including the covariates in each model, entering all the independent variables in the model in the same step. A sensitivity analysis was made for investigating the effect of including the individuals (n = 10) who had gone through radiation therapy as part of their treatment. In order to avoid a Type 1 error a Bonferroni correction was applied, and the corrected Bonferroni α 0.05 and 11 regression analysis run per dependent variable, the Bonferroni corrected α was set to 0.005. All analyses were made with SPSS version 24.

## Results

In all, 227 YA ALL survivors responded to the questionnaire and fulfilled the inclusion criteria. They were between 23 and 41 years old when they responded. In all, 147 did not respond to the questionnaire and 42 were excluded due to presence of mental health problems, disabilities (downs syndrome), emigration or longer stays abroad. There was no difference in current age or age at diagnosis or years since diagnosis between respondents and non-respondents/excluded, between 14 and 27 years had passed since diagnosis for both groups. There was a higher proportion of men among the non-respondents/excluded (Table 1). In all, 70 siblings out of 110 responded to the questionnaire. The included siblings were between 18 and 44 years old.

**Table 1:** Descriptive of respondents and non-respondents/excluded

	Respondents (N = 227)	Non-respondents^b^ (N = 189)	P-value^a^
Age, median (range)	28 (23–41)	28 (23–40)	0.672
Age at diagnosis, median (range)	6 (0–15)	6 (0–15)	0.756
Years since diagnose, median (range)	22(14–27)	23(14–27)	0.657
Radiation therapy (CNSirr) n (%)	10 (4.4)	11 (5.8)	0.512
Gender, female n(%)	115 (50.7)	77 (40.7)	0.043

^a^Statistics from Mann–Whitney U-test and chi-squared test when appropriate.

^b^n = 42 were excluded due to presence of mental health problems, disabilities (downs syndrome), emigration or longer stays abroad; n = 147 did not respond the questionnaire

### Characteristics of adult ALL survivors and their siblings

There was a higher proportion of females among the siblings, than the responding YA ALL survivors. A difference between the groups were seen also for occupational status and level of physical activity. There was no difference between the groups regarding age, marital status, proportion of who had children of their own, education, and BMI. Among the YA ALL survivors, 14 (6%) had a cardio-vascular disease (CVD—1 had myocardial infarction, 3 heart failure, 6 stroke and 4 both heart failure and stroke), none of the siblings had a CVD. No differences were seen between the groups for other diseases or health problems investigated (Table 2).

**Table 2:** Characteristics of YA ALL survivors and siblings

	N	YA ALL survivors	N	Siblings	P-value
*Age, median (range)*	227	28 (23–41)	70	28 (18–44)	0.560
*Gender, female, n (%)*	227	115 (51)	70	45 (64)	0.046
*Marital status, n (%)*	224		70		0.414
Married or cohabitant		137 (61)		39 (56)	
In a relationship		20 (9)		10 (14)	
Single		67 (30)		21 (30)	
*Having (any) children, n (%)*	226	69 (31)	70	24 (34)	0.728
*Education*	226		70		0.272
Compulsory school (9-yrs)		12 (5)		2 (3)	
Upper seconday school (2–3 years)		109 (48)		27 (39)	
University < 3 yrs		29 (13)		9 (13)	
University > 3 yrs		76 (34)		32 (46)	
*Occupational status, n (%)*	226		70		0.040
Student		36 (16)		17 (24)	
Working		15 (70)		45 (64)	
Unemployed		16 (7)		1 (1)	
On sick-leave/disability pension		6 (3)		0	
On parental leave		9 (4)		6 (9)	
Other		2 (1)		1 (1)	
*Monthly Income (€), n (%)*	223		68		0.334
< 1000		61 (27)		18 (27)	
1000–1 900		40 (18)		9 (13)	
2000–2 900		92 (41)		25 (37)	
3000–3 900		21 (9)		12 (18)	
4000–4 900		7 (3)		4 (6)	
≥ 5000		2 (1)		0 (0)	
*Physical training, n (%)*	226		70		< 0.001
Inactive		73 (32)		8 (11)	
Active		100 (44)		49 (70)	
Very active		53 (24)		13 (19)	
*BMI, n (%)*	202		58		0.684
< 18.5		4 (2)		0	
19–24		114 (56)		36 (62)	
25–29		57 (28)		16 (28)	
30–34		18 (9)		5 (9)	
> 34		9 (5)		1 (2)	
*Comorbidity, n (%)*					
CVD	226	14 (6)	68	0 (0)	< 0.05
Respiratory disease	226	19 (8)	69	8 (12)	0.422
Headache/migrane	215	77 (36)	70	28 (40)	0.528
Ulcer disease	225	3 (1)	69	0 (0)	0.335
Diabetes	226	3 (1)	70	0 (0)	0.333
Renal disease	209	8 (4)	70	2 (3)	0.491
Connective tissue disease	210	5 (2)	70	0 (0)	0.193
Cancer	212	6 (3)	69	1 (1)	0.513
Liver disease	215	2 (1)	70	0 (0)	0.418

### HRQoL in adult ALL survivors, siblings and in a normative population

**Fig. 1:**
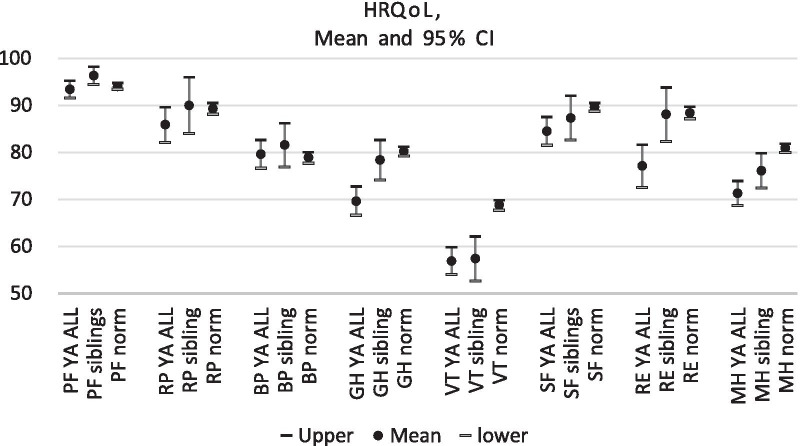
Mean and 95% confidence intervals of included HRQoL parameters, in YA ALL survivors, siblings and normative values from the general population. PF = Physical Functioning; RP = Role Physical; BP = Bodily Pain; GH = General Health; VT = Vitality; SF = Social Functioning; RE = Role Emotional; MH = Mental Health

The YA ALL survivors rated their general health and their role emotional significantly lower than the siblings whereas the YA ALL survivors scored lower on social functioning and role emotional than what was found in normative values. Both YA ALL survivors and the siblings scored lower on vitality and mental health than normative values (Fig. 1).

The Mann Whitney U-test showed significant differences between YA ALL survivors and the siblings in the SF-36 subscales general health (p = 0.010) and in role emotional (p = 0.023). All differences remained in the sensitivity analysis excluding the 10 individuals who had gone through radiation therapy, (general health p = 0.017; role emotional, p = 0.024).

### Depression, anxiety and stress in YA ALL survivors and siblings

The distribution in depression, anxiety and stress, imply that the YA ALL survivors were more likely to respond “extremely severe” depression, anxiety and stress, and less likely to respond “normal” than the siblings. However, the difference between the YA ALL survivors and the siblings regarding depression, anxiety and stress were non-significant (Table 3).

**Table 3:** Depression, anxiety and stress in YA ALL survivors and siblings

	YA ALL survivors N = 227	Siblings N = 70	*p-value*
*Depression n (%)*			0.146
Normal	154 (69)	57 (84)	
Mild	23 (10)	3 (4)	
Moderate	27 (12)	4 (6)	
Severe	8 (4)	3 (4)	
Extremely severe	10 (5)	1 (2)	
*Anxiety n (%)*			0.668
Normal	178 (80)	57 (84)	
Mild	11 (5)	3 (4)	
Moderate	24 (11)	6 (9)	
Severe	1 (0)	1 (2)	
Extremely severe	10 (5)	1 (2)	
*Stress n (%)*			0.242
Normal	175 (79)	59 (86)	
Mild	21 (10)	3 (4)	
Moderate	15 (7)	3 (4)	
Severe	6 (3)	4 (6)	
Extremely severe	5 (2)	0yn	

### Potential buffering factors and HRQoL, depression, anxiety and stress

There was no difference in mean social support (2.79 vs. 2.89; p = 0.501) or general self-efficacy (30.55 vs. 30.97; p = 0.539) between YA ALL survivors and siblings. However, a higher scoring in social support and higher self-efficacy were both significantly associated with better HRQoL (all dimensions), and lower depression, anxiety and stress in YA ALL survivors. Social support had strongest association with vitality, depression and mental health. General self-efficacy had strongest association with mental health, vitality and general health (Table 4). All results from the regression analysis including YA ALL survivors were significant.

**Table 4:** Regression analysis of association between HRQoL parameters and social support and self-efficacy in YA ALL survivors, adjusted for age and gender

	Social support			General self-efficacy		
	β^a^	*p*	Adjusted R^2^	β^a^	*p*	Adjusted R^2^
*HRQoL dimensions*						
Physical function	0.236*	< 0.001	0.061	0.299*	< 0.001	0.077
Role physical	0.244*	< 0.001	0.065	0.294*	< 0.001	0.073
Bodily pain	0.266*	< 0.001	0.074	0.221*	0.001	0.035
General health	0.341*	< 0.001	0.122	0.367*	< 0.001	0.123
Vitality	0.425*	< 0.001	0.181	0.408*	< 0.001	0.150
Social functioning	0.343*	< 0.001	0.123	0.370*	< 0.001	0.125
Role emotional	0.289*	< 0.001	0.088	0.290*	< 0.001	0.070
Mental health	0.356*	< 0.001	0.132	0.425*	< 0.001	0.168
*Mental health parameters*						
Depression	− 0.374*	< 0.001	0.154	− 0.361*	< 0.001	0.130
Anxiety	− 0.222*	0.001	0.067	− 0.275*	< 0.001	0.088
Stress	− 0.222*	0.001	0.075	− 0.312*	< 0.001	0.109

*p < 0.005; ^a^ Standardized beta coefficient

Among the siblings, higher social support was associated with better HRQoL regarding the dimensions general health, vitality, role emotional, mental health and depression (p ≤ 0.005) whereas self-efficacy was significantly associated with the HRQoL dimensions general health, and mental health. Social support showed highest association with vitality, depression and role emotional. Better self-efficacy showed highest association with general health and mental health (Table 5).

**Table 5:** Presenting results from the regression analysis of association between HRQoL parameters and the potentially buffering factors (social support and self-efficacy) in siblings

	Social support			General self-efficacy		
	β^a^	*p*	Adjusted R^2^	β^a^	*p*	Adjusted R^2^
*HRQoL dimensions*						
Physical function	0.096	0.444	− 0.017	− 0.164	0.181	0.028
Role physical	− 0.017	0.892	− 0.026	− 0.106	0.389	0.012
Bodily pain	0.148	0.243	− 0.005	0.152	0.219	0.024
General health	0.368*	0.003	0.104	0.369*	0.003	0.131
Vitality	0.472*	< 0.001	0.183	0.281	0.025	0.075
Social functioning	0.331	0.007	0.082	0.224	0.067	0.050
Role emotional	0.424*	< 0.001	0.157	0.079	0.519	0.007
Mental health	0.421*	0.001	0.145	0.340*	0.005	0.113
*Mental health parameters*						
Depression	− 0.464*	< 0.001	0.205	− 0.277	0.023	0.107
Anxiety	− 0.125	0.341	0.001	− 0.135	0.291	0.044
Stress	− 0.265	0.039	0.051	− 0.208	0.097	0.068

Age and gender adjusted

*p < 0.005; ^a^Standardized beta coefficient

## Discussion

The results from this study show that the YA ALL survivors scored significantly lower on the HRQoL parameters general health and role emotional, than a control group consisting of their siblings. The general health score from SF-36 primarily assess perceived physical health, rather than mental health [33] whereas the role emotional score reflects role impairment due to emotional distress. Low role emotional scores may thus represent an increased vulnerability to poor outcome in working life [34] and an increased risk of sickness absence [17]. The YA ALL survivors also reported significantly lower general health, vitality, social functioning, role emotional and mental health than normative values from the general population. This is in line with the results from a French study, where ALL survivors scored lower on social functioning and role emotional than the general population, however that study had a wide age range of participants, including both younger adults and children [35].

In this study, both YA ALL survivors and the siblings reported lower vitality (higher fatigue) and worse mental health than the general population [27], which imply that fatigue and mental health are important factors to observe in both groups. Low vitality, or fatigue, includes both physical and mental fatigue and has high impact on the quality of life and disease burden in individuals with chronic conditions [36, 37] and is highly correlated with depression [38]. Scoring low vitality has also shown to be a predictor for chronic widespread pain [39]. It should be noted however, that the normative values from the general population are based on data assessed in the early 1990’s [27] whereas data for this study was assessed in 2012, and some of the deviation from the normative values may thus reflect a cultural or societal change during this time period.

The YA ALL survivors seemed less likely to have “normal levels” of symptoms of depression, anxiety and stress, than the siblings, although no statistical differences were seen between the groups. It was almost twice as common among YA ALL survivors compared to the siblings to report moderate to extremely severe depression, however the difference was not significant. A similar trend was seen for scoring moderate to extremely severe anxiety whereas levels of distress in YA ALL survivors and siblings were comparable. A prevalence for reporting depression, anxiety and stress in YA ALL survivors has been shown in studies from other contexts [10] and in comparison with siblings [40]. However, the levels of anxiety and distress found in our study were lower and were based on self-report rather than parent report.

A significant association between self-efficacy and all the HRQoL parameters as well as the mental health parameters were found among the YA ALL survivors. The strongest associations were seen for general health, vitality, social functioning, mental health, depression and stress. Among the siblings however, self-efficacy was associated only with the HRQoL parameters general health and mental health. The findings are supported by previous research investigating quality of life and self-efficacy in adult cancer patients [41, 42]. Social support showed to be of importance among both YA ALL survivors and the siblings regarding general health, vitality, mental health and depression. The associations were somewhat stronger among the siblings than among the YA ALL survivors. The R^2^ values were low overall in all associations found in the regression analysis, indicating that social support and self-efficacy (respectively) only explain a smaller part of the variability of the HRQoL parameters, depression, anxiety and stress.

In summary, the differences and similarities between YA ALL survivors and their siblings, regarding HRQoL, and the associations between the buffering factors and HRQoL and mental health parameters suggests that (1) self-efficacy may be of greater importance as a buffering factor among adults who have had pediatric ALL than among the siblings; (2) the siblings rate poorer vitality and mental health than the norm, suggesting an increased vulnerability to ill health than the norm; (3) social support is potentially a more important buffering factor for poor mental health and low vitality than self-efficacy among the siblings. Previous studies support our finding of lower HRQoL among YA ALL survivors, and the importance of self-efficacy as a potential buffering factor. However, our study adds knowledge about the importance of the buffering factors in relation also to the siblings.

Between 14 to 27 years had passed since diagnose among the YA ALL survivors included in this study and they were at the time of the study 23 to 41 years old. Even though many years had passed, differences in some of the health parameters, and differences in the importance of buffering factors were seen between the YA ALL survivors and the siblings. These results recognize the relevance of developing and providing support to these individuals long-time after the end of treatment and even into adulthood to promote their health outcomes and successful introduction to adult life.

A strength of this study is that all participants were adults when this follow-up was conducted. Most other studies do not have this long-term perspective but focus on HRQoL in younger survivors, where shorter period of time has passed from diagnosis [9, 13, 35, 43]. Further, the group consist of survivors among whom a mere part had received radiation therapy (only 4.4% had radiation therapy). The results from this study adds knowledge on how YA ALL survivors may be vulnerable to factors related to ill health also in their adult life. Another strength of this study was the possibility to relate the HRQoL of YA ALL survivors not only to a group of siblings, but also to normative values from a Swedish population, thereby allowing for comparisons to the HRQoL in the general population that is not potentially affected by experiences from growing up together with a brother or sister with a serious illness.

There are however also a few limitations with this study that needs to be addressed. Due to practical reasons for data collection, the siblings were much fewer than the YA ALL survivors which complicates the comparison between the two groups and the possibility to reach significance in the regression analysis. Further, in a cross-sectional study like this, the results can not reveal if any of the factors investigated have any causative relations or predictive value. Future longitudinal studies should further investigate the buffering effect self-efficacy and social support and their relevance as predictors of long-term health outcomes and causal relationships.

## Conclusion

The results from this study suggest that buffering factors, like social support and self-efficacy, may play an important role for psychosocial outcomes and HRQoL among YA ALL survivors later in life and that this group could benefit from continuous support in adult life to handle consequences of their pediatric disease. The study highlights the importance of further studies with long-term follow-up of psychosocial outcomes and HRQoL among YA ALL survivors and investigation of potential buffering and predictive effects of self-efficacy and social support.

## Data Availability

The datasets used and/or analysed during the current study are available from the corresponding author on reasonable request.

## Abbreviations

ALL: Acute lymphatic leukemia
YA: Young adult
HRQoL: Health-related quality of life
